# Draft Genome Sequence of *Xanthomonas translucens* pv. graminis Pathotype Strain CFBP 2053

**DOI:** 10.1128/genomeA.01174-15

**Published:** 2015-10-08

**Authors:** Céline Pesce, Stéphanie Bolot, Edwige Berthelot, Claude Bragard, Sébastien Cunnac, Marion Fischer-Le Saux, Perrine Portier, Matthieu Arlat, Lionel Gagnevin, Marie-Agnès Jacques, Laurent D. Noël, Sébastien Carrère, Ralf Koebnik

**Affiliations:** a UMR Interactions Plantes Micro-organismes Environnement (IPME), IRD-Cirad-Université Montpellier, Montpellier, France; b Earth and Life Institute, Applied Microbiology Phytopathology, Université Catholique de Louvain, Louvain-la-Neuve, Belgium; c INRA, Laboratoire des Interactions Plantes Micro-organismes (LIPM), UMR 441, Castanet-Tolosan, France; d CNRS, LIPM, UMR 2594, Castanet-Tolosan, France; e UMR 1345, Institut de Recherche en Horticulture et Semences (IRHS), INRA, SFR, Beaucouzé, France; f CIRM, CFBP, Collection Française de Bactéries associées aux Plantes, INRA, IRHS, Angers, France; g Université Paul Sabatier, Toulouse, France; h UMR Peuplements Végétaux et Bioagresseurs en Milieu Tropical (PVBMT), CIRAD, Saint-Pierre, La Réunion, France

## Abstract

Strains of *Xanthomonas translucens* pv. graminis cause bacterial wilt on several forage grasses. A draft genome sequence of pathotype strain CFBP 2053 was generated to facilitate the discovery of new pathogenicity factors and to develop diagnostic tools for the species *X. translucens*.

## GENOME ANNOUNCEMENT

Forage grasses, such as bluegrass (*Poa* spp.), bromegrass (*Bromus* spp.), fescue (*Festuca* spp.), oat grass (*Arrhenatherum elatius*), orchardgrass (*Dactylis glomerata*), quack grass (*Agropyron repens*), ryegrass (*Lolium* spp.), and timothy (*Phleum pratense*), are major crops that serve to feed livestock throughout the world. Some grasses, such as fescue, smooth brome, and crested wheatgrass, are also commonly used as turf in landscape gardening and for sports grounds. In addition, grasses are of economic interest for revegetation of dumped or burned areas. Last, but not least, different grasses serve the survival of wildlife, for example, as nesting sites of birds, as cover for small animals, as a habitat for foraging raptors, or simply to feed all kinds of animals that depend on grass leaves, shoots, roots, or seeds as a source of calories.

All of the above-mentioned grass species can be infected by different pathovars (pvs. arrhenatheri, cerealis, graminis, phlei, phleipratensis, and poae) of *Xanthomonas translucens*. Recently, we sequenced the genome of *X. translucens* pv. cerealis pathotype strain CFBP 2541, which was isolated from *Bromus inermis* (1). To gain further insight into the pathogenicity of grass-pathogenic xanthomonads, we then sequenced another genome of an *X. translucens* pv. graminis strain (2). *X. translucens* pv. graminis can invade the plant via wounds (e.g., after feeding or mowing), followed by colonization of the protoxylem lacunae and of the adjacent xylem parenchyma (3). Upon entry into the xylem vessels, the pathogen can spread throughout the leaf, leading to symptoms such as wilting of leaves and necrosis of the entire plant (3).

Pathotype strain CFBP 2053 (ATCC 29091, LMG 726, NCPPB 2700), which was isolated from *D. glomerata* in Switzerland in 1973, was sequenced using the Illumina HiSeq 2000 platform (GATC Biotech, Germany). The shotgun sequencing yielded 18,569,445 read pairs (14,855,556 100-bp paired-end reads, with an insert size of 250 bp, and 3,713,889 50-bp mate-pair reads, with an insert size of 3 kb). A combination of Velvet (4), SOAPdenovo, and SOAPGapCloser (5) yielded 48 contigs ≥500 bp (*N*_50_, 126,255 bp), with the largest contig being 386,541 bp, for a total assembly size of 4,340,322 bp, corresponding to 691× coverage. Contigs were scaffolded into two pseudomolecules using *Xanthomonas euvesicatoria* strain 85-10 as a reference sequence (6).

With these characteristics, our new genome sequence is of much better quality than the first *X. translucens* pv. graminis genome sequence, which consists of 788 contigs (*N*_50_, 8,376 bp) (2). The genome was found to encode a noncanonical hypersensitive response and pathogenicity (Hrp) type III protein secretion system, the genetic organization of which corresponds to that of other *X. translucens* strains (1, 2, 7). Based on the catalog of *Xanthomonas* type III effectors (http://www.xanthomonas.org), strain CFBP 2053 has the same set of type III effectors as the other sequenced strain of this pathovar, except for *avrBs2*, which is present in ART-Xtg29 but not in CFBP 2053.

### Nucleotide sequence accession numbers.

This whole-genome shotgun project has been deposited in DDBJ/EMBL/GenBank under the accession number LHSI00000000. The version described in this paper is the first version, LHSI01000000.
